# Neurological update: use of cardiac troponin in patients with stroke

**DOI:** 10.1007/s00415-020-10349-w

**Published:** 2020-12-29

**Authors:** Jan F. Scheitz, Helena Stengl, Christian H. Nolte, Ulf Landmesser, Matthias Endres

**Affiliations:** 1 Klinik für Neurologie mit Experimenteller Neurologie, Charité-Universitätsmedizin Berlin, Berlin, Germany; 2 Center for Stroke Research Berlin, Charité-Universitätsmedizin Berlin, Berlin, Germany; 3 German Center for Cardiovascular Research (Deutsches Zentrum Für Herz-Kreislaufforschung; DZHK), partner site Berlin, Berlin, Germany; 4 Berlin Institute of Health, Berlin, Germany; 5 Department of Cardiology, Charité-Universitätsmedizin Berlin, Campus Benjamin Franklin, 12203 Berlin, Germany; 6 German Center for Neurodegenerative Diseases (Deutsches Zentrum Für Neurodegenerative Erkrankungen; DZNE), partner site Berlin, Berlin, Germany

**Keywords:** Ischemic stroke, Cardiac biomarker, Stroke-heart-syndrome, Cardiac troponin, Brain–heart interaction, Myocardial injury, Myocardial infarction

## Abstract

Cardiac troponin is a specific and sensitive biomarker to identify and quantify myocardial injury. Myocardial injury is frequently detected after acute ischemic stroke and strongly associated with unfavorable outcomes. Concomitant acute coronary syndrome is only one of several possible differential diagnoses that may cause elevation of cardiac troponin after stroke. As a result, there are uncertainties regarding the correct interpretation and optimal management of stroke patients with myocardial injury in clinical practice. Elevation of cardiac troponin may occur as part of a ‘Stroke-Heart Syndrome’. The term ‘Stroke-Heart Syndrome’ subsumes a clinical spectrum of cardiac complications after stroke including cardiac injury, dysfunction, and arrhythmia which may relate to disturbances of autonomic function and the brain–heart axis. In this review, we provide an up-to-date overview about prognostic implications, mechanisms, and management of elevated cardiac troponin levels in patients with acute ischemic stroke.

## Introduction

Current guidelines on the early management of stroke recommend measuring cardiac troponin (cTn) in all patients with suspected stroke [1]. The reason for this recommendation is to improve early detection of a cardioembolic source of stroke and to identify patients with high risk of poor outcomes and heart disease [2]. However, there are no specific recommendations about how to interpret and manage stroke patients with elevated cTn. Recent evidence suggests that concomitant or preceding acute coronary syndrome (ACS) is only one of several possible causes of cTn elevation after stroke [3, 4]. Myocardial injury, as detected by cTn, may occur as a direct consequence of stroke, which has been termed as ‘Stroke-Heart syndrome’ [3]. Since the clinical presentation of an ACS may be atypical after stroke and because treatment of suspected ACS may entail a relevant bleeding risk, there remains a clinical dilemma for stroke physicians. In this review, we provide an up-to-date overview about frequency, prognostic utility, potential mechanisms, and management of elevated cTn levels in patients with acute ischemic stroke.

## Cardiac troponin, myocardial injury and myocardial infarction

The troponin complex—which consists of troponin T, troponin I and troponin C—is essential for muscle contraction of skeletal and cardiac muscle cells [5]. Troponins T and I have cardiac-specific isoforms and are thereby well suited as specific biomarkers for myocardial injury. Because of the clear superiority compared to other (“historical”) markers like CK-MB, the introduction and clinical establishment of cTn in 1989 markedly changed recommendations for the diagnosis and treatment of myocardial infarction [6]. Since 2010, with advances in assay technologies, high-sensitivity cTn assays (hs-cTn) were introduced [7]. Troponin assays are classified as being of “high-sensitivity” when they are able to detect cTn in > 50% of a reference group of apparently healthy individuals with a coefficient of variation of < 10% at the 99th percentile upper reference limit (URL) in this reference group [8]. Hs-cTn assays provide an improved sensitivity and are capable of identifying a relevant proportion of patients who had undetectable cTn concentrations with conventional cTn assays [8]. This leads to a higher accuracy in the early detection of acute myocardial infarction [9, 10]. Although cTn is highly specific to identify and quantify myocardial injury, it is not specific for the clinical diagnosis of myocardial infarction or cardiac ischemia [10, 11]. In fact, with improved sensitivity of cTn assays, the specificity for acute myocardial infarction as the underlying reason for elevation of hs-cTn is further reduced. For this reason, the 2018 Fourth Universal Definition of myocardial infarction makes a clear distinction between ‘myocardial injury’ and ‘myocardial infarction’ (see Info-Box). While elevated hs-cTn above the assay-specific 99th percentile URL represents evidence of myocardial injury, serial measurements with evidence of a rise/fall pattern (> 20% change) allow differentiation between acute and chronic myocardial injury. The diagnosis of acute myocardial infarction is, however, restricted to patients with evidence of myocardial ischemia as the underlying cause of their acute myocardial injury. Evidence of myocardial ischemia is documented by: clinical symptoms of myocardial ischemia, changes in ECG (new ischemic changes or a development of pathological Q waves), evidence in diagnostic imaging of a new loss of viable myocardium or a new regional wall motion abnormality or the detection of a coronary thrombus by angiography or autopsy [10]. The most relevant types of myocardial infarction for stroke neurologists are type 1 MI due to acute coronary atherothrombosis, and type 2 MI due to a mismatch of oxygen demand/supply by pathophysiological mechanisms other than atherothrombosis (Type 2 MI, Info-Box) [10].

Info-Box

**Table:** 

Myocardial injury	Presence of elevated cardiac troponin value above the assay-specific 99th percentile upper reference limit (URL)
Chronic myocardial injury	Myocardial injury but no acute change in serial measurement
Acute myocardial injury	Myocardial injury with rise or fall (> 20%) in serial measurement
Acute myocardial infarction	Acute myocardial injury with clinical evidence of acute myocardial ischemia: - Symptoms of myocardial ischemia (e.g. chest pain) - New ischemic ECG changes - Development of pathological Q waves - Imaging evidence of new loss of viable myocardium or new regional wall motion abnormality in a pattern consistent with an ischemic etiology; Identification of a coronary thrombus by angiography or autopsy
Type 1 myocardial infarction	Myocardial infarction caused by atherothrombotic coronary artery disease and usually precipitated by atherosclerotic plaque disruption (rupture or erosion)
Type 2 myocardial infarction	Myocardial infarction caused by a mismatch between oxygen supply and demand by a pathophysiological mechanism other than coronary atherothrombosis (e.g. tachyarrhythmia, hypotension or shock, severe anemia)
Acute non-ischemic myocardial injury	Myocardial injury in the absence of an ischemic cause (i.e. no evidence of myocardial ischemia as described above in myocardial infarction)
Takotsubo Syndrome (TTS)	Acute but mostly reversible heart failure syndrome that can mimic myocardial infarction. TTS is often triggered by a preceding emotionally or physically stressful event. The pathophysiologic mechanism is thought to be a strong sympathetic stimulation with overshooting catecholamine levels. Over 90% of patients with TTS are postmenopausal women
Stroke-heart syndrome	Evidence of acute myocardial injury, cardiac dysfunction, or cardiac arrhythmia within 30 days after acute ischemic stroke with peak within 72 h and potential long-term cardiac sequelae. Cardiac symptoms are either newly detected after the ischemic stroke event, or clear evidence shows worsening of cardiac function after stroke. Needs to be differentiated from type 1 MI and systemic causes of non-ischemic myocardial injury

## Cardiac troponin in stroke—frequency and prognostic implications

With the advent of highly-sensitive cTn measurements in the emergency setting, it is becoming increasingly recognized that myocardial injury (i.e. cTn elevation) is frequent in patients presenting with several medical emergencies including acute stroke [12]. Data from observational studies with consecutive patients with acute ischemic stroke demonstrate that 30–60% of patients have elevated cTn levels upon hospital admission, when contemporary high-sensitivity cardiac troponin (hs-cTn) assays are applied [3, 13, 14]. This is higher than would be expected from populations of elderly individuals without acute stroke (~ 15%) [15]. In approximately 15% of patients, cTn elevation even exceeds cut-offs that are used for the triage of patients presenting with chest pain as “suspected myocardial infarction” in the emergency department [16]. Older age, a history of structural and coronary heart disease, and a history of cardiovascular risk factors including impaired kidney function are established risk factors for hs-cTn elevation after stroke [14, 17, 18]. Moreover, stroke-related factors, such as stroke severity and lesion site within the central autonomic network (i.e. the insular cortex), are associated with hs-cTn elevation after stroke [19]. This supports the notion that stroke-induced mechanisms may directly lead to myocardial injury (see paragraph about possible mechanisms). Stroke patients with elevated cTn levels are at high risk of developing cardiac complications that range from transient ECG alterations (including repolarization changes and QTc prolongation) to severe arrythmia and reduced LV function [3, 17, 20].

Several studies provide consistent evidence that myocardial injury in acute ischemic stroke is strongly associated with poor functional outcomes and a more than 2-fold increase in mortality [3, 14, 18]. Beyond the strong association with mortality, recent studies suggest that elevated hs-cTn levels in stroke patients are associated with higher rates of major cardiovascular events [21, 22]. Moreover, hs-cTn levels have also been linked to the severity of cerebral small vessel disease and impaired cognitive function [23, 24]. In a prospective cohort of patients with mild-to-moderate first ever stroke, patients with hs-cTn levels in the highest quartile were 1.8-fold more likely to have cognitive impairment according to Mini-Mental-State-Examination compared to those in the lowest quartile. This association was maintained within the first three years after the index event [24].

## Causes of troponin elevation in stroke

**Fig. 1:**
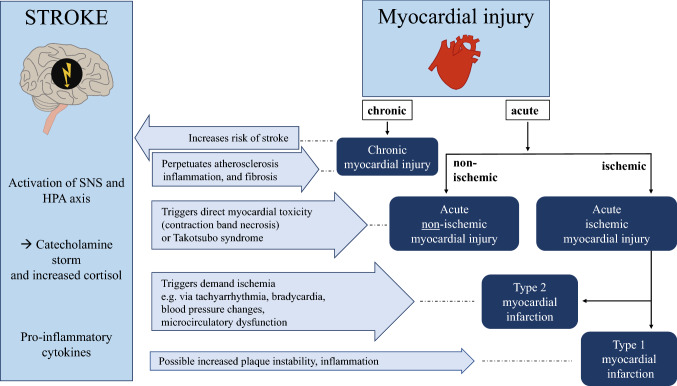
Taxonomy of myocardial injury and possible impact of stroke. Myocardial injury can be acute or chronic. In case of evidence of cardiac ischemia (clinical symptoms, ischemic ECG alterations, evidence of new wall motion abnormalities) myocardial infarction can be suspected (see Info-Box for definitions). Stroke-associated alterations in the central autonomic network lead to autonomic imbalance with activation of the sympathetic nervous system (SNS) and hypothalamic–pituitary–adrenal axis (HPA). In addition, stroke results in a pro-inflammatory response. Thereby, stroke can trigger both ischemic and non-ischemic myocardial injury

A first crucial step to determine the most likely cause of cTn elevation is to apply serial measurements [10, 11]. Acute myocardial injury displays a rise and/or fall pattern of hs-cTn levels (changes > 20%, per definition) [10]. Previous studies indicate that in 85–95% of all stroke patients, and in approximately two thirds of stroke patients with elevated cTn upon hospital admission, serial measurements show no relevant temporal change in cTn levels [14, 25]. This suggests that the majority of stroke patients have chronic rather than acute myocardial injury. In these cases, chronic structural and coronary heart disease are the most likely underlying causes [3]. Chronic myocardial injury is also common in chronic kidney disease which is probably not mainly driven by impaired renal clearance but rather reflects severity of cardiac comorbidities [26, 27]. Importantly, the definitions of temporal change and timing of serial measurements differed in these studies which lead to uncertainty about the true incidence of acute and chronic myocardial injury after stroke. If there is evidence of acute myocardial injury, then myocardial ischemia due to atherothrombotic myocardial infarction (i.e. type 1 MI) needs to be considered. In the small prospective TRoponin ELevation in Acute ischemic Stroke (TRELAS) study, coronary angiography revealed acute coronary “culprit” lesions indicative of type 1 MI in approximately 25% of stroke patients with cTn elevation above of at least 4 times the URL [28]. However, the cause of acute myocardial injury beyond type 1 MI can be diverse and range from myocardial ischemia due to type 2 MI (e.g. demand ischemia) or microcirculatory dysfunction, to non-ischemic conditions, such as takotsubo syndrome, myocarditis, as well as systemic conditions like sepsis or pulmonary embolism (Fig. 1) [10, 11].

In the clinical setting of ischemic stroke, distinct pathophysiological considerations have to be made. Stroke-related autonomic dysregulation with systemic and local cardiac release of catecholamines as well as an enhanced stroke-related systemic inflammatory response may facilitate and perpetuate cardiac damage [3], ([29] accepted for publication October 12, 2020). Recently, a “Stroke-Heart Syndrome” has been defined which summarizes the clinical consequences of this common pathophysiological pathway leading to cardiac manifestations, such as myocardial injury, cardiac dysfunction and arrhythmia shortly after stroke [3], ([29] accepted for publication October 12, 2020). In brief, “Stroke-Heart Syndrome” can be considered a stroke-induced “stress-test” for the heart that may (1) promote coronary demand ischemia (i.e. type 2 MI) via tachyarrhythmia or hypertensive crisis, and microcirculatory dysfunction via endothelial dysfunction or oxidative stress, (2) lead to direct neurocardiogenic myocardial necrosis (contraction band necroses, similar to catecholamine toxicity), and (3) trigger takotsubo syndrome in susceptible individuals, such as elderly women. Thus, stroke may precipitate both ischemic (1) and non-ischemic (2, 3) myocardial injury (Fig. 1). This conceptual framework that brain–heart signals contribute to cTn elevation after stroke is supported by several experimental and clinical studies. Myocardial dysfunction and injury can be induced in murine models of stroke and are associated with cardiac inflammation and fibrosis, especially after selective injury of the murine correlate of the insular cortex [30, 31]. The insular cortex is key regulatory center within the central autonomic network, i.e., a network of brain structures involved in the sympathetic and parasympathetic control of cardiac function in response to emotional and physical stress [3]. Clinical studies suggest that this might also be relevant in humans. By applying voxel-based lesion-symptom mapping, lesions in the right dorsal anterior insular cortex were associated with the magnitude of change in cTn levels after stroke [19]. The TRELAS study demonstrated that in ischemic stroke patients presenting with cTn elevation above diagnostic cut-offs for myocardial infarction, nearly half of these patients had no evidence of obstructive coronary artery disease [28]. Even though this was a small study, these findings suggest that alternative causes of cTn elevation other than type 1 MI are common in the clinical setting of stroke. The notion that stroke may induce and perpetuate cardiac injury is also supported by a large population-based study from Canada showing a 4.5-fold increased risk of major adverse cardiac events in patients with stroke compared with well-matched individuals without stroke. Interestingly, this increased risk is most pronounced within the first 30 days after the event (HR 25.0, 95% CI 20.5–30.5) [32].

## Management of troponin elevation in stroke

**Fig. 2:**
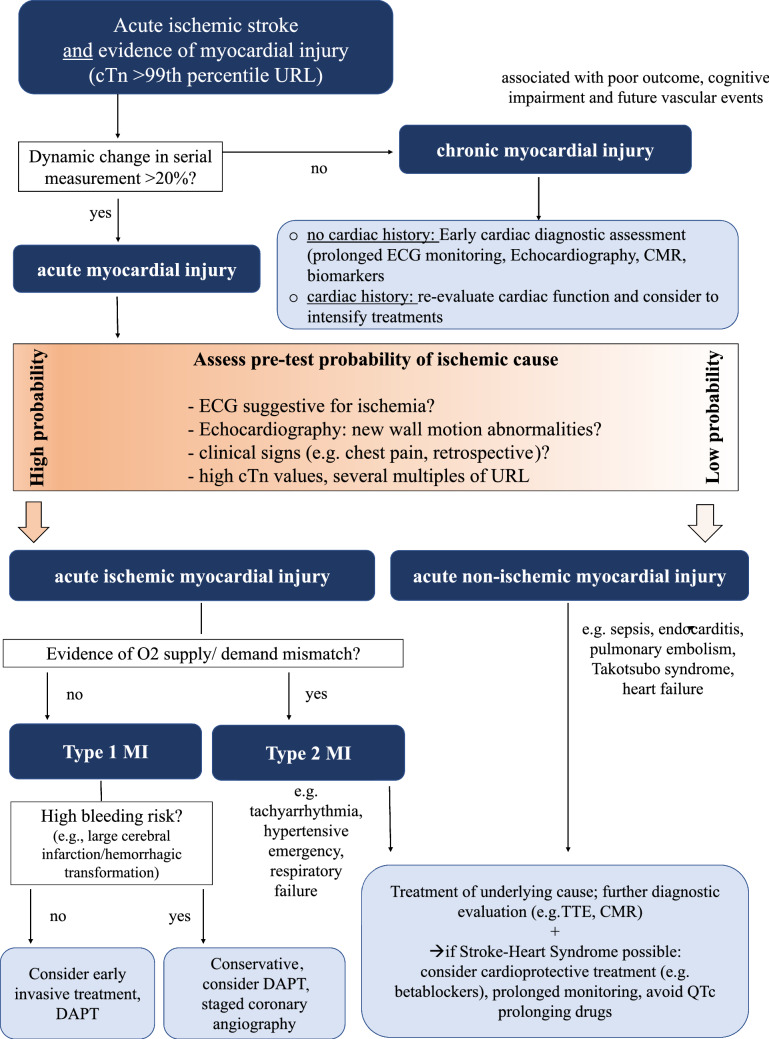
Interpretation and management of troponin elevation after acute ischemic stroke. Systematic approach to the interpretation, classification, and diagnostic steps of elevated cardiac troponin levels in patients with acute ischemic stroke. (Algorithm is based on literature [4, 10, 11] and has not been validated). *CMR* cardiovascular magnetic resonance tomography, *cTn* cardiac troponin, *DAPT* dual antiplatelet therapy, *ECG* electrocardiogram, *MI* myocardial infarction, *SHS* stroke heart syndrome, *TTE* transthoracic echocardiography, *URL* upper reference limit

Until now, there are no clear guideline recommendations about how to diagnose and treat stroke patients with elevated cTn. Most stroke guidelines, however, provide some advice for the general cardiac work-up following an acute stroke [1, 33]. These recommendations include at least a routine 12-channel electrocardiogram, 24 h (better 72 h) of continuous rhythm monitoring, and echocardiography to search for atrial fibrillation and other cardiac embolic sources. In addition, cardiological guidelines make recommendations about diagnostic procedures in patients presenting with acute coronary syndromes [34]. Figure 2 integrates these two perspectives and shows an algorithm that can be used for the management of acute ischemic stroke patients with cTn elevations. We recommend that these patients should be jointly evaluated by both cardiologists and neurologists to enable accurate diagnoses but also avoid unnecessary invasive diagnostic procedures. As emphasized above, the first step is to apply serial measurements to differentiate between acute and chronic myocardial injury. This is particularly relevant since pre-morbid levels of cTn are usually not known. If there is evidence of chronic myocardial injury but no apparent history of cardiac disease, further cardiac evaluation and staging of cardiovascular risk seem to be warranted. Clinical presentation (i.e. typical cardiac symptoms, such as chest pain or dyspnea), thorough evaluation of ECG for the presence of signs of myocardial ischemia, appreciation of absolute cTn levels and the magnitude of their temporal change, and, in case of uncertainty, further non-invasive measures to identify regional wall motion abnormalities (e.g. echocardiography) constitute the basis for the etiological work-up. The role of coronary CT angiography or cardiovascular MRI in patients with stroke needs to be established in future studies, but may be useful in individual patients if there is experience with these diagnostic measures in the respective center. On the other hand, evaluation of the individual risk of secondary brain hemorrhage (e.g. patients with large infarct sizes, multiple cerebral microbleeds or prior intracerebral hemorrhage) needs to be considered to decide whether a conservative or early invasive strategy is pursued. When a more invasive strategy seems acceptable, bleeding risk may be reduced using drug eluting balloons instead of stents and short-term periods of platelet inhibition. There is evidence that diagnostic coronary angiography during the early phase after stroke (i.e. within the first 3–7 days) is feasible and safe [28].

## Troponin and other neurological disease

Myocardial injury is not only seen in ischemic stroke but can occur in in a broad spectrum of acute neurological disorders, especially in severe acute conditions, such as intracerebral hemorrhage, subarachnoid hemorrhage (SAH), status epilepticus or head trauma [3, 32, 35, 36]. Similar to ischemic stroke, in patients with intracerebral hemorrhage, at least mildly elevated cTn levels can be observed in up to 40% of patients and are independently associated with clinical severity and functional outcome [37]. Following SAH, elevated cTn is observed in 20–40% of patients with conventional generation assays which suggest that even higher proportions may have such an abnormality if more sensitive assays are used [35, 38]*.* Elevated cTn levels in SAH patients are associated with higher SAH severity (i.e., higher Hunt and Hess scores), risk of delayed cerebral ischemia, poor functional outcome and death [38, 39]. Since patients with SAH are usually younger and less likely to have premorbid structural heart disease compared with patients with ischemic stroke, neurocardiogenic mechanisms for the development of myocardial injury seem to play an even more important role in this population. This is supported by autopsy studies in patients who died from SAH. Patients who died from SAH are more likely to have contraction band necrosis compared to patients who died from other causes [40]. Contraction band necrosis is a distinct myocardial lesion seen in patients with sympathetic overdrive and has been attributed to a direct catecholamine toxicity [41]. A temporal rise of hs-cTn can also be observed after seizures and has been associated with focal to bilateral-tonic clonic seizure type, longer seizure duration, and higher ictal heart rate [42]. Another study found hs-cTn elevation in 26% of patients with generalized onset seizures [43]. Here, elevated hs-cTn levels were associated with reduced heart rate variability, QTc prolongation, and higher catecholamine levels. Even after other acute neurological conditions that are considered ‘mild’ or ‘benign’, cTn elevation has been described. An intriguing example is transient global amnesia (TGA). TGA is often preceded by an emotionally or physically stressful event leading to focal metabolic disturbance within the hippocampus, which is another region involved in the central autonomic control of the heart [44]. In two recent retrospective cohort studies, the prevalence of a cardiac involvement in TGA patients was investigated [45, 46]. Approximately 25% of TGA patients presented with elevated hs-cTn levels. After multivariable adjustment, the diagnosis of TGA was associated with a higher risk of hs-cTn elevation than diagnosis of transient ischemic attack (TIA), migraine with aura or acute vestibular syndrome [46]. Altogether, these correlations indicate that mechanisms similar to those described for the ‘Stroke-Heart syndrome’ are involved in the occurrence of myocardial injury after acute neurological conditions.

## Open questions

More data are needed to determine the proportion of stroke patients with cTn elevation that eventually need invasive diagnostic and therapeutic measures. The multicenter, observational PRAISE study (PRediction of acute coronary syndrome in acute ischemic StrokE; NCT03609385) has recently completed recruitment. PRAISE was designed to provide data from diagnostic coronary angiography to identify predictors of the acute coronary syndrome after ischemic stroke [47]. The findings of PRAISE will help to refine the clinical algorithms presented in this manuscript. On the other hand, more research is needed to quantify the impact of neurocardiogenic mechanisms in cTn elevation after stroke, and to identify therapeutic targets to prevent stroke-induced heart injury. The ongoing CORONA-IS study (CardiOmyocyte injuRy follOwiNg Acute Ischemic Stroke; NCT03892226) applies multi-parametric cardiovascular MRI and autonomic ECG markers to determine the association between autonomic dysfunction and myocardial tissue alterations associated with myocardial injury after stroke. Finally, the long-term consequences of cTn elevation after stroke need to be scrutinized in prospective longitudinal studies. Given the increasingly recognized role of cardiac biomarkers for individual stratification of vascular risk, the potential role of cTn to guide clinical decision-making with regard to secondary prevention measures deserves further study.

## Conclusion

cTn elevation—indicative of acute or chronic myocardial injury—is frequently observed in patients with ischemic stroke. The majority of patients display no temporal change of cTn elevation in serial measurements and therefore have chronic myocardial injury. There is strong evidence that both acute and chronic cTn elevation are associated with an increased risk of severe cardiac complications, short-term and long-term mortality, and probably also an increased risk of future cardiovascular events. Acute elevation in cTn levels should prompt timely diagnostic evaluation and may occur due to concomitant myocardial infarction but also as part of a ‘Stroke-Heart Syndrome’ based on neurocardiogenic mechanisms. Interdisciplinary collaborations of stroke neurologists and cardiologists are needed to guide reasonable management of stroke patients with elevated cTn.
