# Correction to: Neurological update: use of cardiac troponin in patients with stroke

**DOI:** 10.1007/s00415-021-10434-8

**Published:** 2021-02-25

**Authors:** Jan F. Scheitz, Helena Stengl, Christian H. Nolte, Ulf Landmesser, Matthias Endres

**Affiliations:** 1 Klinik für Neurologie mit Experimenteller Neurologie, Charité-Universitätsmedizin Berlin, Berlin, Germany; 2 Center for Stroke Research Berlin, Charité-Universitätsmedizin Berlin, Berlin, Germany; 3 German Center for Cardiovascular Research (Deutsches Zentrum Für Herz-Kreislaufforschung; DZHK), partner site Berlin, Berlin, Germany; 4 Berlin Institute of Health, Berlin, Germany; 5 Department of Cardiology, Charité-Universitätsmedizin Berlin, Campus Benjamin Franklin, 12203 Berlin, Germany; 6 German Center for Neurodegenerative Diseases (Deutsches Zentrum Für Neurodegenerative Erkrankungen; DZNE), partner site Berlin, Berlin, Germany

## Correction to: Journal of Neurology https://doi.org/10.1007/s00415-020-10349-w

The original version of this article unfortunately contained a mistake. Reference [23] has to be deleted.

The original article has been corrected.

